# Phthalate exposure and risk of ovarian dysfunction in endometriosis: human and animal data

**DOI:** 10.3389/fcell.2023.1154923

**Published:** 2023-07-25

**Authors:** Huan Yi, Huamin Wu, Wenbin Zhu, Qi Lin, Xiaoyan Zhao, Rong Lin, Yan Luo, Lixiang Wu, Danmei Lin

**Affiliations:** ^1^ National Key Gynecology Clinical Specialty Construction Unit of China, Fujian Provincial Key Gynecology Clinical Specialty, Fujian Provincial Maternity and Children’s Hospital, Affiliated Hospital of Fujian Medical University, Fuzhou, Fujian, China; ^2^ Department of Obstetrics and Gynecology, Maternal and Child Health Hospital of Fuzhou, Fuzhou, Fujian, China; ^3^ Fujian Health College, Health Management Department, Fuzhou, Fujian, China

**Keywords:** environmental endocrine disruptors, endometriosis, DEHP, ovarian function, steroid synthesis

## Abstract

**Objective:** We aimed to explore the correlations between and possible mechanisms of common environmental endocrine disruptors, phthalates, and ovarian dysfunction in endometriosis.

**Methods:** Subjects were included in the case group (*n* = 107) who were diagnosed with endometriosis by postoperative pathology in Fujian Maternal and Child Hospital from February 2018 to February 2021, and the women who were excluded from endometriosis by surgery were as the control group (*n* = 70). The demographic information of the subjects were evaluated by questionnaire, and the clinical characteristics were evaluated by medical records and 3-year follow-up results. Gas chromatography‒mass spectrometry was used to quantify 10 metabolites of phthalates, including dimethyl ortho-phthalate (DMP), mono-n-methyl phthalate (MMP), dioctyl ortho-phthalate (DEP), mono-ethyl phthalate (MEP), di-n-butyl ortho-phthalate (DBP), mono-butyl phthalate (MBP), benzylbutyl phthalate (BBzP), mono-benzyl; phthalate (MBzP), diethylhexyl phthalate (DEHP) and mono-ethylhexyl phthalate (MEHP), in the urine samples of the subjects. Furthermore, a total of 54 SD rats were exposed to DEHP 0, 5, 50, 100, 250, 500, 1,000, 2000, and 3,000 mg/kg/day for 2 weeks. The SD rats’ body weight, oestrus cycle changes, and serum anti-mullerian hormone (AMH) levels were evaluated. After sacrifice, the mass index of the rat uterus and bilateral ovaries were calculated. Finally, bioinformatics analysis of rat ovarian tissues was performed to explore the possible mechanism. SPSS 24.0 (IBM, United States) was used for data analysis. *p*-value <0.05 was considered statistically significant.

**Results:** The human urinary levels of DMP (*p* < 0.001), MMP (*p* = 0.001), DEP (*p* = 0.003), MEP (*p* = 0.002), DBP (*p* = 0.041), MBP (*p* < 0.001), BBzP (*p* = 0.009), DEHP (*p* < 0.001), and MEHP (*p* < 0.001) were significantly higher in women with endometriosis than in controls. Notably, DEHP was a significant risk factor for endometriosis (OR: 11.0, 95% CI: 5.4–22.6). The area under the ROC curve increased when multiple phthalates were diagnosed jointly, reaching 0.974 as the highest value, which was helpful for the diagnosis of endometriosis. *In vivo* experiments showed that after DEHP exposure in rats, the mass index of the ovary and uterus decreased in a dose-dependent manner; the oestrus cycle of SD rats was irregularly prolonged and disordered; and the serum AMH level was negatively correlated with the DEHP exposure dose (Rho = −0.8, *p* < 0.001). Bioinformatics analysis of rat ovarian tissues showed that seven genes involved in the steroid biosynthesis pathway were upregulated and may play a negative role in ovarian function.

**Conclusion:** Exposure to phthalates, especially DEHP, is associated with the occurrence of endometriosis and affects women’s reproductive prognosis and ovarian function. The steroid biosynthesis pathway may be related to ovarian dysfunction. The detection of phthalate in urine may become a new biological target for the diagnosis of endometriosis.

## Introduction

Endocrine-related diseases have increased worldwide (Sirohi et al., 2021). Endometriosis is one of the most common diseases in gynaecology, which is characterized by a chronic inflammatory process and proliferation dependent on estrogen, mainly affecting pelvic tissues, such as ovaries (Bulun et al., 2019). Endometriosis can be divided into four types according to the lesion site, namely, endometrioma, peritoneal endometriosis, deep invasive endometriosis and other types of endometriosis, such as abdominal incision endometriosis, pulmonary endometriosis and ureteral endometriosis, etc. The local inflammatory environment caused by endometrioma will induce fibrosis in ovarian cortex, reduce the number of primordial follicles and mature oocytes, and adversely affect ovarian reserve (Donnez et al., 2016; Sanchez et al., 2017). After removing part of the ovaries from women with endometrioma, it may cause iatrogenic damage and further decrease the ovarian reserve (Yılmaz Hanege et al., 2019). The effect of peritoneal endometriosis on ovarian reserve is still controversial. Deep invasive endometriosis and other types of endometriosis have less definite effects on ovarian reserve than endometrioma, and there is still a lack of relevant research. Approximately 176 million women worldwide suffer from this disease, accounting for 10%–15% of women of childbearing age, and its incidence is increasing yearly (Sirohi et al., 2021). Progesterone, oral contraceptives and gonadotropin-releasing hormone agonists can antagonize the effect of estrogen, thus inhibiting the development of endometriosis, but it cannot be completely cured (Vercellini et al., 2014). Even after standardized surgical treatment, chronic pelvic pain, postoperative decrease in ovarian reserve and infertility caused by endometriosis can’t be completely improved, which seriously affects the women’s quality of life (Endometriosis Committee et al., 2018; Yılmaz Hanege et al., 2019).

In recent years, many studies have shown that the occurrence of endometriosis is related to the exposure level of certain endocrine disrupting chemicals (EDCs). Phthalates, commonly known as “plasticizers”, are ubiquitous environmental endocrine disruptors that exist in plastic products through noncovalent bonds, so they easily enter the human body through dietary intake, inhalation, and skin contact (Dutta et al., 2020). Phthalates exist mainly in the form of DEHP in the human living environment, such as in soil, groundwater, rivers, lakes, seas, cosmetics, beverages, food packaging and decoration materials. Exposure to such chemicals has toxic effects on the reproductive system, leading to a series of gynaecological endocrine-related diseases, such as polycystic ovary syndrome, endometriosis, spontaneous abortion, primary premature ovarian failure, and even malignant tumours (Encarnação et al., 2019).

Scholars from all over the world have not stopped studying the correlation between EDCs and endometriosis or the mechanism. Studies have shown that bisphenol A, phthalates, and organic chlorides (e.g., dioxins, dioxin-like compounds, organochlorine pesticides, and polychlorinated biphenyls) are significantly related to the prevalence of endometriosis (Sirohi et al., 2021). The metabolites of phthalates, such as DEHP, MEHP, DBP, BBzP and MBzP, can be effectively detected in the urine or blood of endometriosis patients (Reddy et al., 2006; Upson et al., 2013; Pednekar et al., 2018). Phthalates can imitate the effect of oestrogen, which may affect the occurrence and development of endometriosis by changing the conduction of related signalling pathways (Sirohi et al., 2021).

However, the definite association between endometriosis and EDCs is unclear in both humans and rats. Therefore, we assessed the association between endometriosis and phthalates in human and animal data. Moreover, bioinformatics analysis was used to explore the correlation and possible mechanism of phthalates exposure and endometriosis ovarian dysfunction.

## Methods

### Population and study design

Women who were confirmed to have endometriosis by histopathological analysis of laparoscopic surgery in Fujian Provincial Maternity and Children’s Hospital from February 2018 to February 2021 were selected as the case group. In the same period, women who underwent abdominal surgery for benign gynaecological diseases or tubal ligation without endometriosis were used as controls. The exclusion criteria were infectious diseases such as hepatitis; chronic diseases such as hypertension, diabetes, and kidney disease; uterine fibroids, pelvic inflammatory disease, gynaecological tumours, pregnancy or other obstetrical diseases; or history of consuming sex hormone drugs or food. The demographic information of the subjects involved was evaluated by questionnaire, including age, body mass index, medical insurance, pregnancy and parity, eating habits and exposure to plastic products and cosmetic products. Clinical characteristics were evaluated by medical records and 3-year follow-up results, including visual analogue scores (VAS) for dysmenorrhea before and after surgery, infertility, clinical pathological classification, disease stage, recurrence and postoperative pregnancy.

The research protocol was approved by the hospital ethics committee (Ethics review document: 2,020,001), and all the research subjects signed informed consent forms. Researchers conduct animal experiments humanely and strictly abide by the ethical regulations of animal experiments.

### Urinary phthalate measurements

Ten millilitres of morning urine was collected from the study subjects, 2 mL was taken for the urine specific gravity test, and the remaining liquid was centrifuged at 3,000 rpm for 10 min at room temperature. The supernatant was stored in a refrigerator at −20 degrees. The batch was thawed at room temperature for testing. Sample collection and storage materials (urine cups, pipettes, centrifuge tubes, cryotubes) were all quantitatively tested for phthalate metabolites to eliminate inspection errors. A total of 10 phthalate metabolites were detected by gas chromatography‒mass spectrometry (GC‒MS) (Table 1).

**TABLE 1:** Parent phthalate diesters and corresponding urinary phthalate metabolites.

Parent phthalate diesters	Primary hydrolysed monoester metabolite
Dimethyl ortho-phthalate (DMP)	Mono-n-methyl phthalate (MMP)
Dicthyl ortho-phthalate (DEP)	Mono-ethyl phthalate (MEP)
Di-n-butyl ortho-phthalate (DBP)	Mono-butyl phthalate (MBP)
Benzylbutyl phthalate (BBzP)	Monobenzyl phthalate (MBzP)
Diethylhexyl phthalate (DEHP)	Mono-ethylhexyl phthalate (MEHP)

Parameter settings of the Agilent 6890-GC/MS: An HP-5 capillary column (30 m × 0.25 mm lD, 0.25 µm, Agilent, United States) was selected, hydrogen was used as the carrier gas, the flow rate was 1 mL/min, the inlet temperature was 230°C, and the sample volume was 1 μL. The initial temperature of the column was 100°C for 1 min, the temperature was programmed to 200°C at 10°C/min and then to 250°C at 6°C/min for 3 min, and the transmission line temperature was 250°C. The ion source was an EI source, the temperature was 250°C, and the electron bombardment energy was 70 eV. The solvent delay time was 5.8 min, qualitative determination was carried out in full-beating and scanning mode, and the scanning range was 50–500 m/z. In brief, the detection steps included water bath enzymolysis, solid phase extraction, elution, drying the eluent with high purity nitrogen, derivatization and sample analysis. The detection data of each metabolite were corrected by its urine specific gravity, namely, metabolite content/urine specific gravity. When the measured level is lower than the limit of detection (LOD) (detection threshold/radix 2) was substituted in the statistics.

### Observation on animals exposed to DEHP

A total of 54 clean-grade SD female rats (healthy, sexually mature, not pregnant), 6–7 weeks old, weighing approximately 200 g, were selected from Wu’s Experimental Animal Center (Animal Quality Licence: SCXK (Fujian) 2016–0,002). Rats were bred in a barrier environment, with laminar ventilation, a room temperature of 22–26C, day and night lighting, an alternating light/dark time of 10/14 h, humidity at 50%–70%, and food and water *ad libitum* (according to the China Laboratory Animal Environment and Facilities Standard GB14925.2010).

The rats were randomly divided into 9 groups (*n* = 6), and then adapted to the environment and reared for 2 weeks. The safety limit value of DEHP recommended by China Food Safety Risk Assessment Expert Committee is 3.0 mg/d, for example, if a person weighs 60 kg, then 0.05 mg/kg/d is the lowest dose; considering the uncertainty coefficient of 100, the lowest exposure dose of rats is about 100 times of human exposure, and it is recommended to take 5 mg/kg/day orally (Kay et al., 2013). For repeated dose toxicity study, 2 weeks of administration time is enough to detect ovarian toxicity caused by DEHP (Takai et al., 2009). In our experiment, each group was exposed to a DEHP concentrations of 0, 5, 50, 100, 250, 500, 1,000, 2000, or 3,000 mg/kg/day by garaging once every morning from 8 a.m. to 9 a.m. for 2 weeks. As a colorless and odorless oily liquid, corn oil is similar to DEHP, so it becomes the vehicle of our experiment. The weight of each rat was measured daily with an electronic scale (accurate to 0.1 g), and vaginal exfoliated cells were collected to observe changes in the oestrus cycle. Before and after the experiment, 1–2 mL of rat venous blood was collected and centrifuged at 3,000 rpm for 10 min at room temperature, and the supernatant was taken and stored at −80°C. Serum AMH levels were measured with an automatic biochemical immunoassay. After 2 weeks of continuous exposure to DEHP, rats were euthanized during dioestrusl under anesthesia. The rat’s uterus and ovaries were weighed (accurate to 0.0001 g), and the mass index was calculated (uterine or ovarian mass (g)/rat body mass (g)).

Considering that the actual exposure dose of DEHP in the population is lower than its safe limit value, the 5 mg/kg/day group is the closest to the actual dose of the population, which is more suitable for further study on the influence and possible mechanism of DEHP exposure on ovarian function. Three samples of rat ovarian tissue were randomly selected from the control group and the 5 mg/kg/day group for transcriptome sequencing. We used DESeq to analyze the difference of gene expression. The volcanic map of differentially expressed genes was drawn by ggplots2 software package in R language. Two-way cluster analysis was carried out on the union of differential genes and samples of all comparison groups by using R language Pheatmap software package. Kyoto Encyclopedia of Genes and Genomes (KEGG) enrichment analysis was used to find related pathways and upregulated genes.

### Immunohistochemical (IHC) analysis

The 4 μm-thick sections of ovary and uterus of rats were dewaxed and rehydrated in ethanol and water. Antigen retrieval was performed in citrate buffer (pH 6.0, 15 min). Tissue sections were blocked with 5% goat serum (ELAB, China). The sections were incubated for 2 h at 37°C with the primary antibodies, Anti-Squalene Epoxidase Rabbit Polyclonal Antibody (1:100, HUABIO, China) or Anti-CYP51A1/CYP51 (1:50, Abcam, United Kingdom). Sections were then incubated with biotinylated secondary antibody and counter-stained with hematoxylin. Finally, sections were dehydrated and fixed with neutral resin. The percentage of positive cells and staining intensity were observed in images taken at ×400 magnification by two independent investigators blinded to this experiment.

### Quantitative real-time polymerase chain reaction (qPCR)

RNA was extracted from rat ovarian samples by liquid nitrogen grinding and silicon matrix adsorption column. The extracted RNA was subjected to reverse transcription using HiScript first strand cDNA Synthesis Kit (R211-01/02, Vazyme). Real-time fluorescence quantitative PCR (bio-rad, United States) was used for detection. The fluorescent dye of real-time PCR was AceQ qPCR SYBR Green Master Mix (Jizhen Bio, China). The primer sequences used to amplify each gene are shown in Table 2. The qPCR reaction was carried out in 45 cycles of 95°C for 20s and 60°C for 20s. Glyceraldehyde-3-phosphate dehydrogenase (GAPDH) was selected as the reference gene. The relative mRNA levels were relatively quantified via 2^−ΔΔCT^.

**TABLE 2:** The primers information of quantitative real-time PCR.

Gene	Forward sequence (5′-3′)	Reverse sequence (5′-3′)	PCR Products (bp)
GAPDH	GGCTCTCTGCTCCTCCC	CCGTTCACACCGACCTT	96
SQLE	TCT​GGG​AGA​TGC​GTA​TAA​CCT​G	CGT​TCA​CAA​CAA​AGG​AAT​GGC	188
CYP51	GTT​AGA​AAT​CGT​TTA​GGG​GAG​C	ATC​TTT​ACG​CAC​AGA​AGT​GGC	194
RT1-CE2	CCT​TCT​CCA​TCC​ACC​GAC​TC	GTT​TCT​CCT​CCT​CAC​AAT​AGC​C	123

### Statistical analyses

SPSS 24.0 (IBM, United States) was used for data analysis. Measurement data with a non-normally distribution are expressed as quartiles and were compared by means of the Kruskal‒Wallis and Mann‒Whitney U tests. Measurement data conforming to a normal distribution are expressed as the mean ± standard deviation (X ± S) and were compared by means of the *t*-test. The Ξ^2^ test and Fisher exact test were used for counting data. The analysis of disease risk factors was performed with univariate and multivariate logistic regression analyses, using odds ratios (ORs) and 95% confidence intervals (CIs). The best critical value was determined by ROC curve analysis. The relationship between serum AMH and DEHP exposure was analysed by Spearman correlation analysis and linear regression analysis. *p*-value <0.05 was considered statistically significant.

## Results

### Clinical features of participants

A total of 107 patients with endometriosis served as the case group. The average age was 33.6 ± 6.7 years. During the same period, 70 healthy women served as a control group, and their average age was 32.6 ± 6.8 years. Compared to controls, the use of plastic products (*p* = 0.046) and cosmetics (*p* = 0.005) was significantly higher among the cases with endometriosis. Infertility (*p* = 0.011) and dysmenorrhea (*p* < 0.001) were increased in the endometriosis group compared with the control group (Table 3).

**TABLE 3:** General characteristics of study participants.

Characteristic	Cases (*n* = 107) n (%)	Controls (*n* = 70) n (%)	$t/x^{2}$	*p*-value
Maternal age (years)	33.6 ± 6.7	32.6 ± 6.8	0.949	0.344
			(Cohen’s d: 0.148)	
Age categories (years)			0.5	0.925
17–24	5 (4.7)	3 (4.3)		
25–34	54 (50.5)	39 (55.7)		
35–44	41 (38.3)	24 (34.3)		
≥45	7 (6.5)	4 (5.7)		
BMI (kg/m^2^)	21.0 ± 2.7	20.9 ± 2.4	0.284	0.777
			(Cohen’s d: 0.039)	
Underweight (<18 .5)	21 (19.6)	14 (20.0)	0.8	0.945
Normal (18.5–23.9)	75 (70.1)	49 (70.0)		
Overweight (24–27.9)	9 (8.4)	6 (8.6)		
Obese (28–29.9)	1 (0.9)	1 (1.4)		
Severe obesity (30–39.9)	1 (0.9)	0 (0)		
Medical insurance			10.1	0.071
Basic Insurance for Urban Employees	49 (45.8)	29 (41.4)		
Basic Insurance for Urban Residents	28 (26.1)	22 (31.4)		
New Rural Cooperative Medical Insurance	19 (17.8)	16 (22.9)		
Business insurance	0 (0)	1 (1.4)		
Own expense	1 (0.9)	2 (2.9)		
Other	10 (9.4)	0 (0)		
Use of plastic products			8.0	0.046
Often	13 (12.2)	2 (2.9)		
Sometimes	21 (19.6)	8 (11.4)		
Occasionally	36 (33.6)	32 (45.7)		
Rarely	37 (34.6)	28 (40.0)		
Cosmetic use			12.6	0.005
Often	33 (30.8)	8 (11.4)		
Sometimes	11 (10.3)	7 (10.0)		
Occasionally	25 (23.4)	31 (44.3)		
Rarely	38 (35.5)	24 (34.3)		
History of infertility			0.9	0.011
Yes	26 (24.3)	6 (8.6)		
No	77 (72.0)	57 (81.4)		
Unknown (deny sex life)	4 (3.7)	7 (10.0)		
Dysmenorrhea			26.9	<0.001
Yes	67 (62.6)	16 (22.9)		
no	40 (37.4)	54 (77.1)		

Data are presented as the means ±SDs, for continuous variables and n (%) for categorical variables. BMI: body mass index.

**FIGURE 1:**
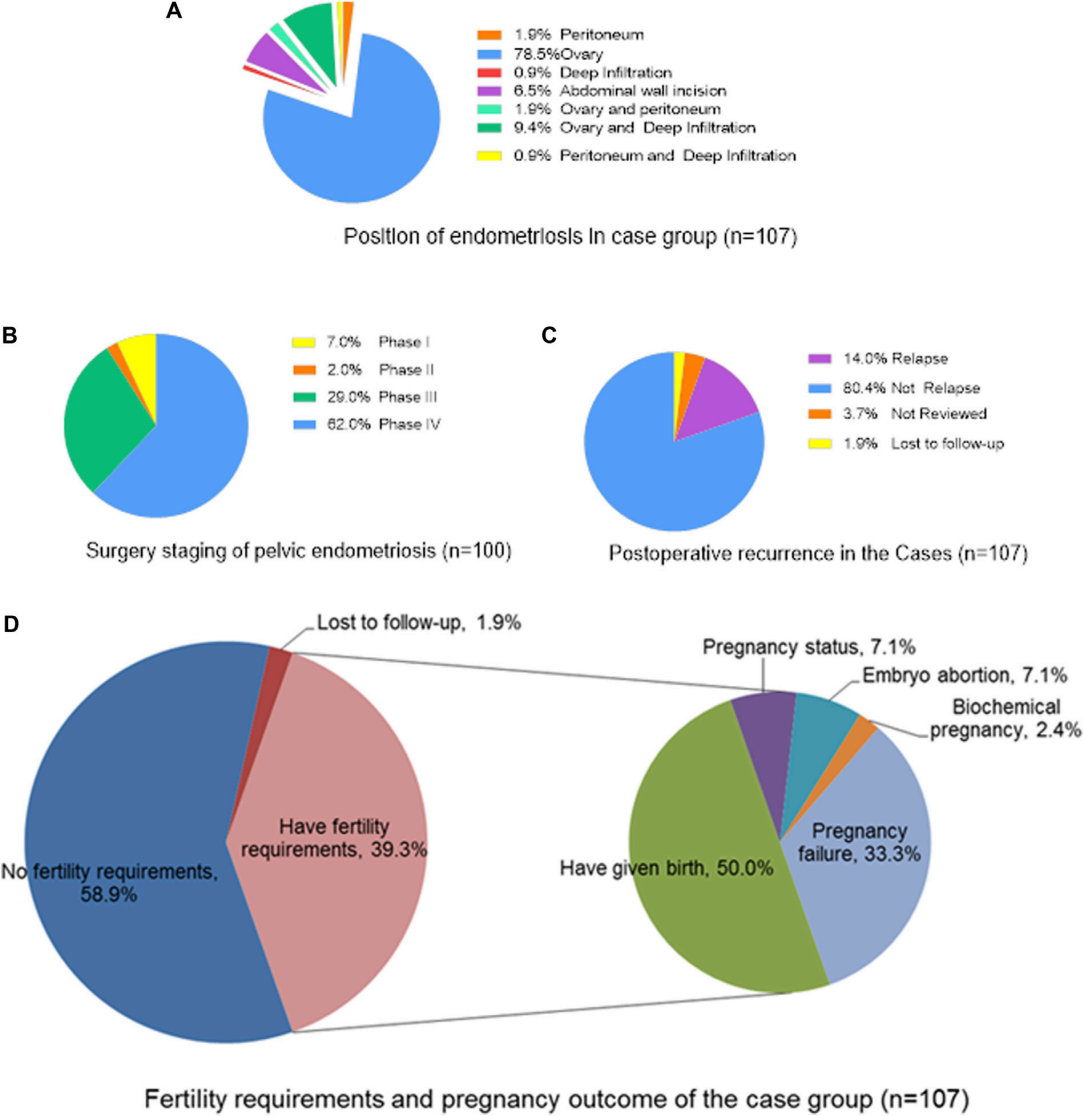
The surgical status and prognostic outcome of the case group (*n* = 107). **(A)** Position of endometriosis in case group (*n* = 107); **(B)** Surgery staging of pelvic endometriosis (*n* = 100); **(C)** Postoperative recurrence in the Cases (*n* = 107); **(D)** Fertility requirements and pregnancy outcome of the case group (*n* = 107).

In this study, except for 7 patients with abdominal incision endometriosis, there were 100 patients with pelvic endometriosis. Furthermore, most of the 100 cases of pelvic endometriosis were stage III (29.0%) or stage IV (62.0%) (according to the 2013 FIGO surgical staging of endometriosis). After 3 years of follow-up, 14.0% of patients had recurrence. Among 42 patients (39.3%) who had fertility requirements, 28 patients (66.7%) had successful pregnancies, 21 (50.0%) successfully delivered (including 15 patients (35.7%) with spontaneous pregnancy and 6 (14.3%) with by IVF-ET), 3 (7.1%) were pregnant (including 1 (2.4%) with spontaneous pregnancy and 2 (4.8%) by IVF-ET), 3 (7.1%) had embryonic abortion (including 1 (2.4%) due to sperm abnormality), and 1 (2.4%) had a biochemical pregnancy (Figure 1). In the control group, 26 people (37.1%) who had fertility requirements, 24 (92.3%) had successful pregnancies, 19 (73.1%) successfully delivered, 4 (15.4%) were pregnant and 1 (3.8%) had embryonic abortion. The success of pregnancy in the control group was significantly higher than that in the case group (*p* = 0.019). In addition, the VAS dysmenorrhea scores before and after the operation were 4.17 ± 3.12 and 1.58 ± 1.77, respectively, and the degree of dysmenorrhea among the postoperative patients was reduced (*p* < 0.001).

### The association between the phthalates content in urine and endometriosis

As shown in Table 4, the detection rates of 10 phthalate metabolites were all greater than 30%. In the case group, the median concentrations of DEHP, MEHP, DMP, MMP, DEP, MEP, DBP and MBP were all higher than their limit of quantitation (LOQ). Among the controls, only the median concentration of DBP was higher than its LOQ. In addition to MBzP (*p* = 0.052), the concentrations of the remaining 9 phthalate metabolites, including DMP (*p* < 0.001), MMP (*p* = 0.001), DEP (*p* = 0.003), MEP (*p* = 0.002), DBP (*p* = 0.041), MBP (*p* < 0.001), BBzP (*p* = 0.009), DEHP (*p* < 0.001) and MEHP (*p* < 0.001), were all significantly higher in the endometriosis group than in the control group.

**TABLE 4:** Laboratory measurement of 10 urinary phthalate metabolites concentrations and distribution.

Phthalate metabolite	LOD (ng/mL)	LOQ (ng/mL)	Study samples (*n* = 177) measured ≥ LOQ n (%)	Cases (*n* = 107) median (IQR)	Controls (*n* = 70) median (IQR)	*p*-value
DMP	0.3	1.0	88 (49.7)	275.3 (0.2–522.9)	0.2 (0.2–0.8)	<0.001
MMP	0.6	2.0	94 (53.1)	46.1 (0.4–118.0)	0.4 (0.4–83.1)	0.001
DEP	5.0	1.7	87 (49.2)	35.7 (3.5–70.6)	3.5 (3.4–30.2)	0.003
MEP	1.0	3.3	84 (47.5)	20.6 (0.7–132.6)	0.7 (0.7–55.6)	0.002
DBP	0.7	2.3	109 (61.6)	79.0 (0.5–786.5)	30.4 (0.5–174.9)	0.041
MBP	1.0	3.3	110 (62.1)	483.5 (40.9–1,441.8)	0.7 (0.7–570.2)	<0.001
BBzP	0.4	1.3	67 (37.9)	0.3 (0.3–96.1)	0.3 (0.3–29.7)	0.009
MBzP	1.1	3.7	73 (41.2)	0.8 (0.8–435.4)	0.8 (0.8–210.0)	0.052
DEHP	0.6	2.0	114 (64.4)	207.3 (118.0–323.6)	<LOD	<0.001
MEHP	0.9	3.0	65 (36.7)	37.5 (0.6–165.4)	<LOD	<0.001

Abbreviations: LOD: limit of detection; LOQ: limit of quantification; IQR: ¼ interquartile range.

**FIGURE 2:**
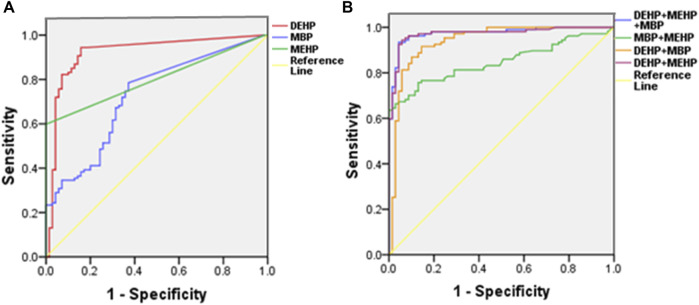
Single-factor **(A)** and multifactor **(B)** ROC curve analysis of DEHP, MEHP, and MBP in urine samples.

The analysis of disease risk factors for the concentration of 10 phthalate metabolites in urine of the cases (*n* = 107) is shown in Table 5. Single logistics analysis found that DMP, MMP, DEP, MEP, DBP, MBP, and DEHP were risk factors for endometriosis (OR: 1.3–8.5). Further multiple logistics analysis found that DEHP (*p* < 0.001) was a significant risk factor for endometriosis. When the DEHP concentration increased by one unit, the risk of disease increased by 11.0 times (95% CI: 5.4–22.6). Table 6 and Figure 2 show the ROC curve and Youden index analysis results of the phthalate metabolites in urine and their correlation with endometriosis. When the concentrations of MBP, MEHP and DEHP in urine were higher than 0.017 mg/L, 0.008 mg/L, and 0.013 mg/L (all above the detection limit), the sensitivity and specificity of the judgement of endometriosis were the highest. The area under ROC curve of MBP is 0.730 (sensitivity: 0.785, 1-specificity: 0.371), which can fairly predict disease diagnosis. The area under ROC curve of MEHP is 0.839 (sensitivity: 0.598, 1-specificity: 0), which is a good prediction of disease diagnosis. Importantly, the area under ROC curve of DEHP is as high as 0.925 (sensitivity: 0.944, 1-specificity: 0.157), which has a very good predictive role in disease diagnosis. In addition, the area under the ROC curve increases when multiple phthalates are diagnosed jointly, reaching 0.974 at the highest.

**TABLE 5:** Single-logistic and multiple-logistic analyses of 10 phthalate metabolites in the case group (*n* = 107).

Phthalate metabolite	Single logistics		Multiple logistics	
	*p*-Value	Odds ratio (95% CI)	*p*-Value	Odds ratio (95% CI)
DMP	<0.001	5.5 (2.8–10.7)	0.061	6.9 (0.9–52.7)
MMP	0.002	1.5 (1.2–1.9)	0.987	1.0 (0.4–2.8)
DEP	0.004	2.5 (1.3–4.6)	1.740	1.7 (0.6–5.3)
MEP	0.010	2.3 (1.2–4.2)	0.370	0.4 (0.1–2.5)
DBP	0.048	1.3 (1.0–1.6)	0.187	0.7 (0.4–1.2)
MBP	<0.001	2.0 (1.5–2.7)	0.082	2.0 (0.9–4.9)
BBzP	0.059	1.9 (1.0–3.6)	—	—
MBzP	0.559	1.2 (0.6–2.2)	—	—
DEHP	<0.001	8.5 (4.8–14.9)	<0.001	11.0 (5.4–22.6)
MEHP	0.997	2.630E9 (0)	—	—

DMP, MMP, DEP, MEP, DBP, MBP, and DEHP, were selected as predictors of multi-variate logistics regression analysis.

**TABLE 6:** ROC curve and Youden index analysis of phthalate metabolites in urine samples of the case group (*n* = 107).

Phthalate metabolite	AUC	SD	*p*-Value	95% CI	Concentration at maximum youden index (mg/L)
DMP	0.653	0.047	0.001	0.561–0.745	0.008
MMP	0.646	0.043	0.001	0.563–0.730	0.004
DEP	0.628	0.044	0.004	0.541–0.714	0.035
MEP	0.635	0.043	0.002	0.550–0.720	0.066
DBP	0.592	0.034	0.039	0.509–0.676	0.423
MBP	0.730	0.039	<0.001	0.654–0.856	0.017
BBzP	0.612	0.045	0.012	0.524–0.077	0.003
MBzP	0.591	0.043	0.042	0.506–0.676	0.293
DEHP	0.925	0.024	<0.001	0.878–0.972	0.013
MEHP	0.839	0.029	<0.001	0.781–0.896	0.008
DEHP + MEHP	0.972	0.012	<0.001	0.949–0.995	—
DEHP + MBP	0.939	0.021	<0.001	0.898–0.979	—
MBP + MEHP	0.847	0.029	<0.001	0.791–0.904	—
DEHP + MEHP + MBP	0.974	0.011	<0.001	0.953–0.995	—

Abbreviations: AUC: area under the curve; SD: standard deviation.

## Effects of DEHP exposure on ovarian function in rats

**FIGURE 3:**
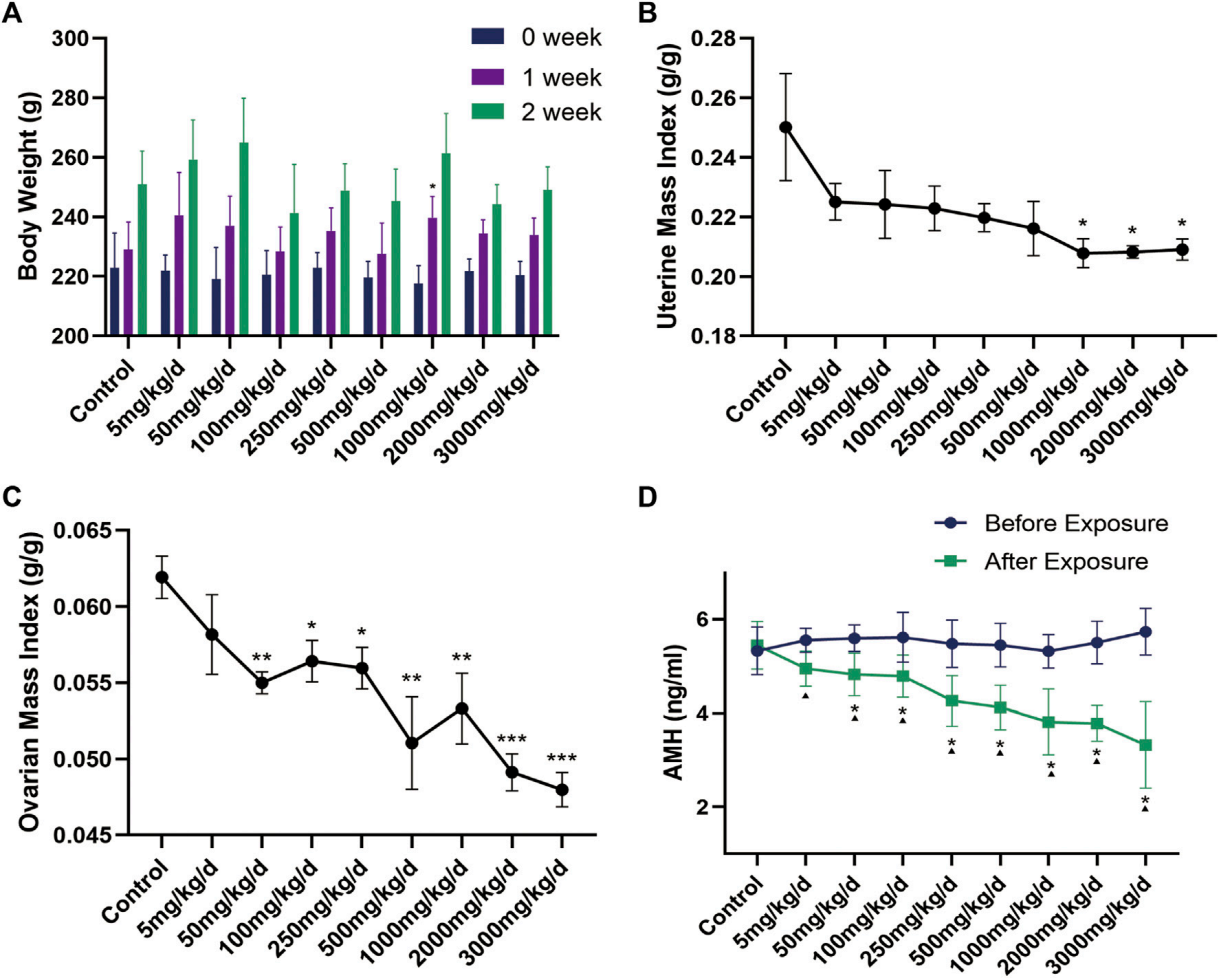
**(A)** Changes in body weight of rats in each group (*n* = 6) during DEHP gavage (g); **(B–C)** Changes in the mass index of the uterus and ovary in rats after DEHP exposure; **(D)** Changes in serum AMH levels in the rats of each group. Note: *: *p* < 0.05, **: *p* < 0.01, ***: *p* < 0.001, *p*-value compared with the control group after the experiment. ▲: *p* < 0.05, *p*-value before and after the experiment in the same treatment group. AMH: anti-Müllerian hormone.

During the 2 weeks of continuous exposure to different doses of DEHP, the appearance of rats is healthy, their fur is clean and bright, and they have no irritating behavior and all survive. There was no reflux and cough in rats during gastric perfusion. There was no significant difference in the initial body weight of rats in each group by repeated measures ANOVA (F = 0.372, *p* = 0.930). After 2 weeks of exposure to different doses of DEHP, there was no significant difference in the weight of rats in each treatment group compared with the control group (*p* > 0.05) (Figure 3A), but the mass index of the ovary and uterus of SD rats was decreased in a dose-dependent manner (Figures 3B,C).

**FIGURE 4:**
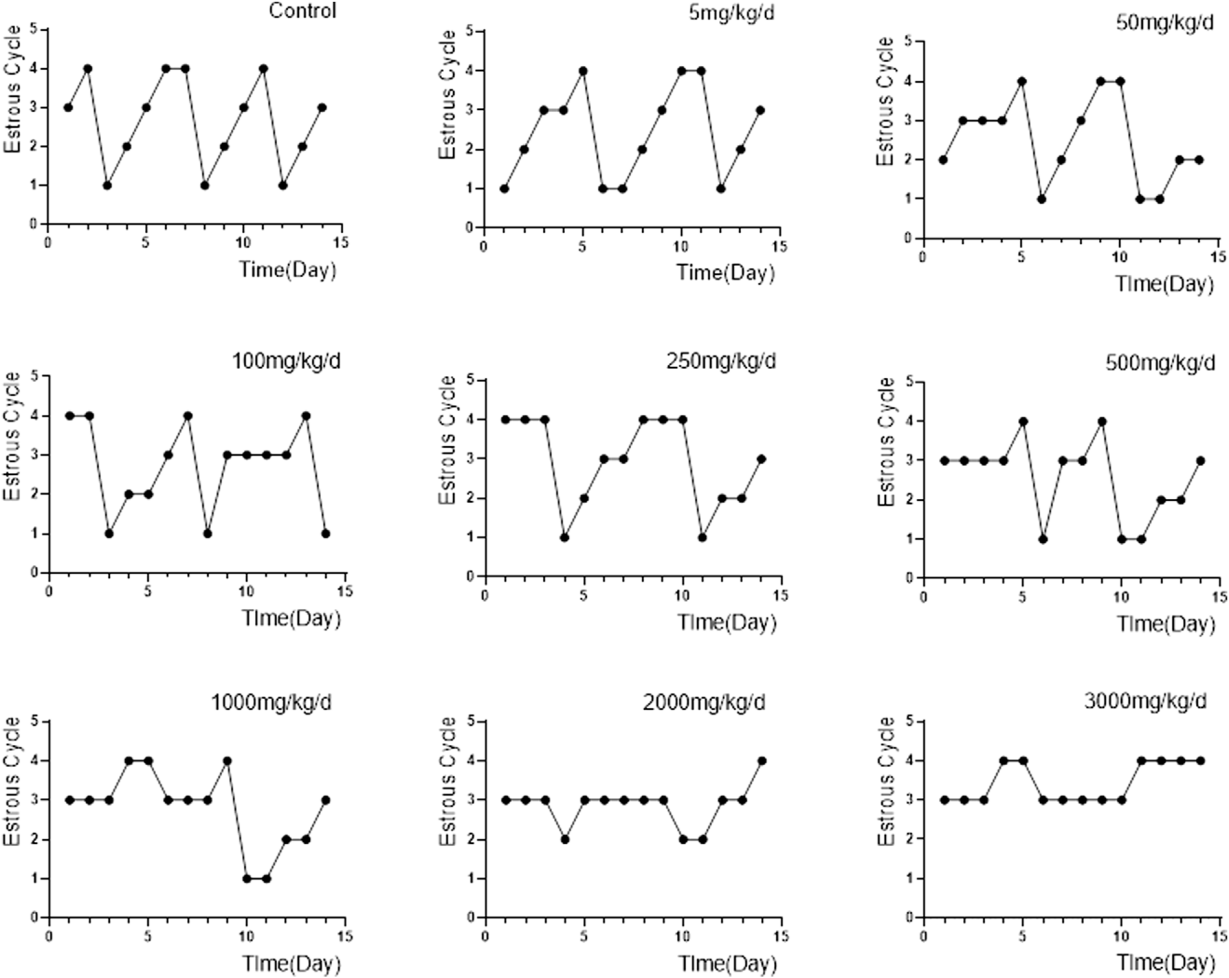
Changes in the oestrous cycle of rats in each group (*n* = 6). Note: “1, 2, 3, 4″in the ordinate represents proestrus, oestrus, meta-oestrus and dioestrus.

The characteristics of each estrous cycle of rats can be accurately identified under the microscope: 1) Proestrus, characterized mainly by nucleated epithelial cells and a few keratinocytes with missing nuclei; 2) Oestrus, characterized by a large number of keratinocytes and a few nucleated epithelial cells; 3) Meta-oestrus, characterized by the presence of keratinocytes, nucleated epithelial cells, and leukocytes; and 4) Dioestrus, characterized by a small number of epithelial cells, a small amount of mucus, and a large number of white blood cells, which look like “gypsophila”. Compared to control group, the 5, 50, 100 and 250 mg/kg/d groups showed irregular prolongation of the oestrus cycle, often delayed in meta-oestrus. The 500, 1,000 and 2000 mg/kg/d groups developed oestrous cycle disturbances, accompanied by irregular prolongation of each phase, mainly in meta-oestrus. The proestrus and oestrus of rats in the 3,000 mg/kg/d groups basically disappeared, and the cycle stagnated mostly in meta-oestrus and dioestrus (Figure 4).

**FIGURE 5:**
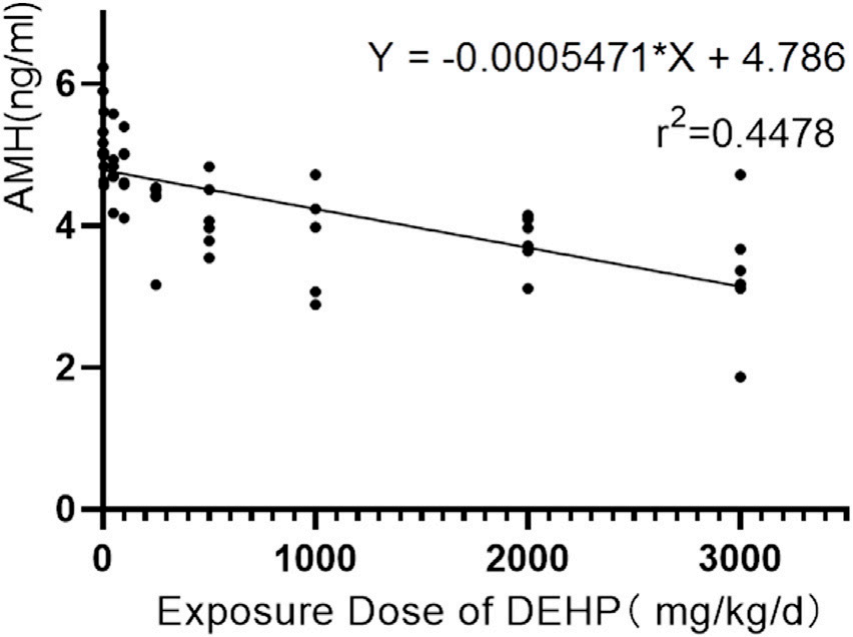
Correlation between serum AMH level and DEHP exposure dose in rats.

Disturbance of the oestrous cycle in rats makes it difficult to accurately assess ovarian function with serum sex hormone levels such as oestradiol and progesterone. In contrast, AMH has many obvious advantages in evaluating ovarian reserve. It is not affected by cyclicity and can reflect the declining trend of ovarian reserve earlier than other traditional biological indicators, like follicle-stimulating hormone, oestradiol and progesterone, which makes it to the first choice for evaluating ovarian reserve (Tsepelidis et al., 2007; Moolhuijsen and Visser, 2020). By univariate analysis of variance, no difference in the pre-experimental AMH levels in each group was found (F = 0.575, *p* = 0.793). With the exception of the 5 mg/kg/d group, the AMH levels of the DEHP exposure groups were significantly lower than those of the control group. Additionally, the AMH levels of the DEHP exposure groups, but not the control group, decreased significantly after DEHP exposure (Figure 3D). In addition, the level of AMH was negatively correlated with the exposure dose of DEHP (Rho = −0.8, *p* < 0.001). The linear regression equation of DEHP exposure on ovarian dysfunction in SD rats is Y = −0.0005471×X + 4.786, *r* ^2^ = 0.4478 (Figure 5).

**FIGURE 6:**
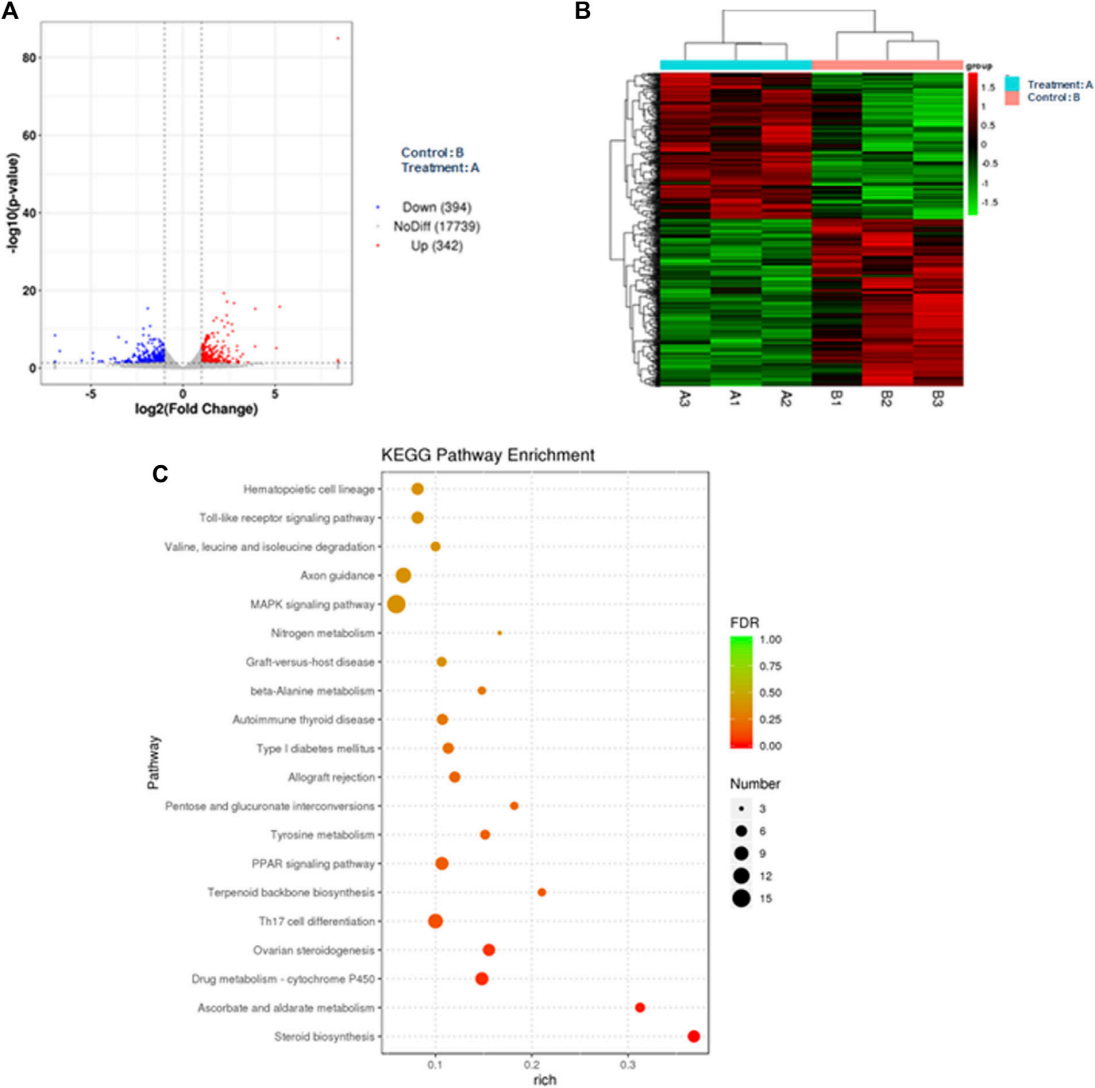
Bioinformatics analysis of ovarian tissue in the control (*n* = 3) and 5 mg/kg/d groups (*n* = 3). Note: **(A)** Volcano map of differentially expressed genes; **(B)** Cluster map of differentially expressed genes; **(C)** KEGG enrichment analysis map.

**FIGURE 7:**
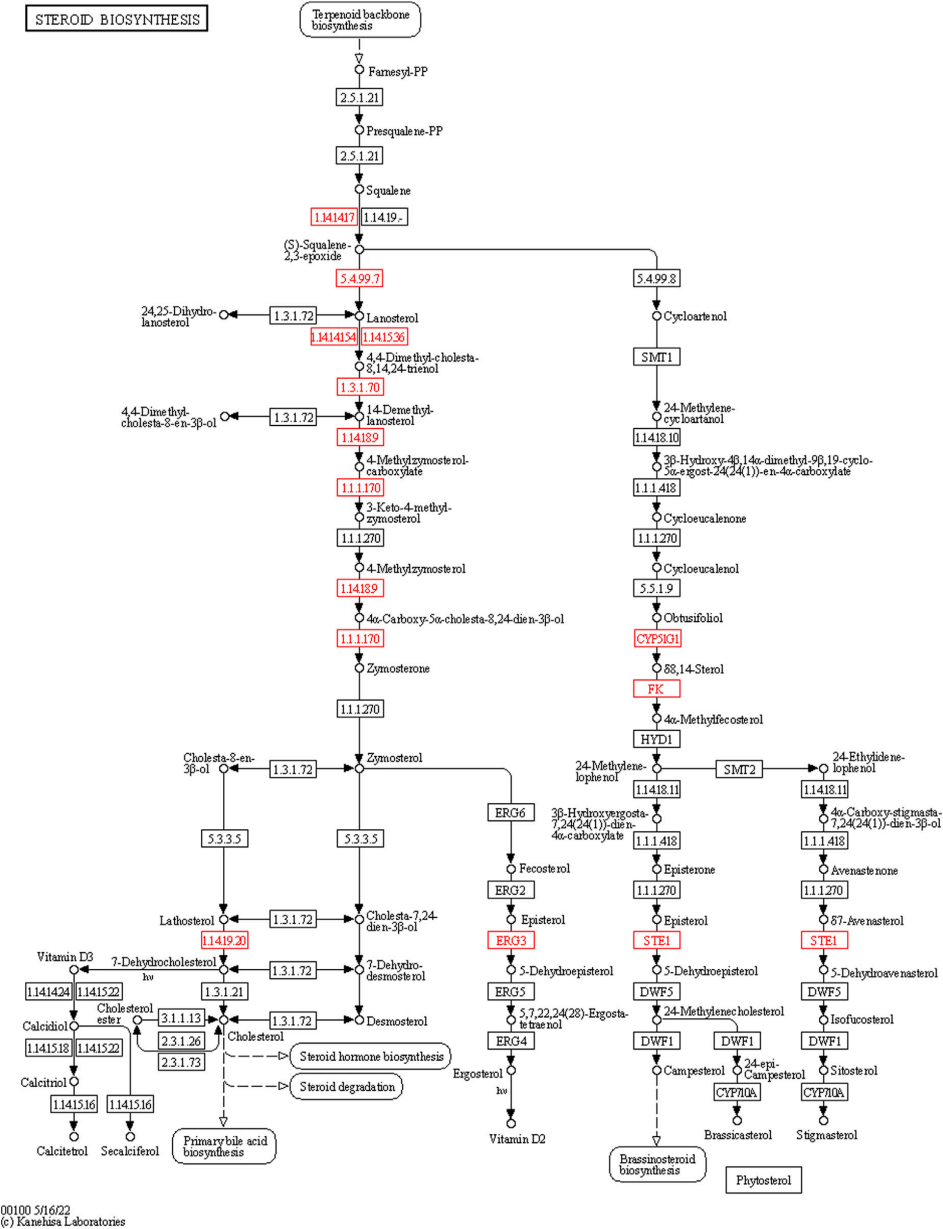
The expression of the steroid biosynthesis pathway genes in the ovarian tissue of the control (*n* = 3) and 5 mg/kg/d groups (*n* = 3). Note: The box represents enzymes, the small circle represents metabolites. The nodes marked in red contain the upregulated genes of the experimental group.

**FIGURE 8:**
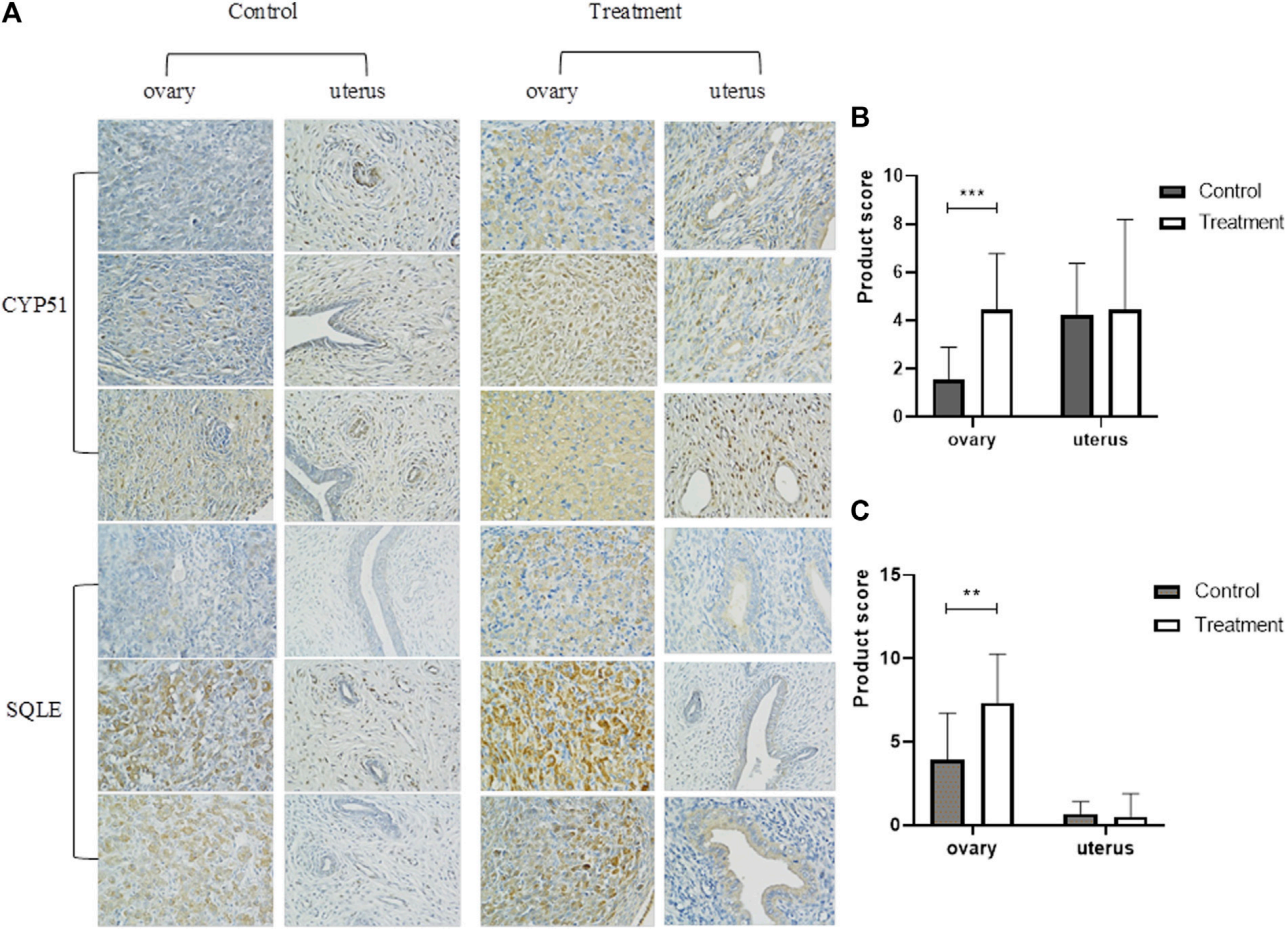
Immunohistochemical determination of ovary and uterus of rats in control group and 5 mg/kg/d treatment group (400×). Note:**(A)** Immunohistochemical staining of uterus and ovary; **(B)**IHC staining of CYP51 expression (400×); **(C)**IHC staining of SQLE expression (400×). **: *p* < 0.01, ***: *p* < 0.001, *p*-value compared with the control group. CYP51: cytochrome P450 family 51; SQLE: squalene epoxidase.

**FIGURE 9:**
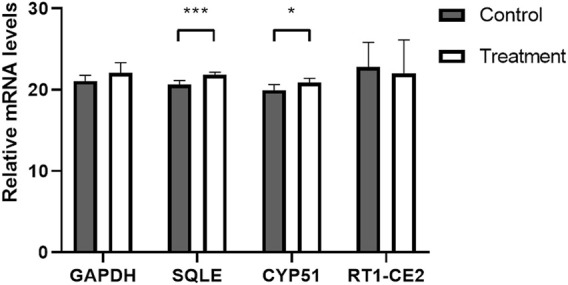
Expression of ovarian related genes in control and 5 mg/kg/d treatment group. Note: *: *p* < 0.05, ***: *p* < 0.001, *p*-value compared with the control group. GAPDH: Glyceraldehyde-3-phosphate dehydrogenase; CYP51: cytochrome P450 family 51; SQLE: squalene epoxidase; RT1-CE2:RT1 class I, locus CE2.

In the bioinformatics analysis, we used DESeq to analyze the differentially expressed genes in the genome “Rattus_norvegicus.Rnor_6.0. dna. topleve. fa”, and the conditions for screening differentially expressed genes were as follows: expression differential multiple |log2FoldChange|>1, and significance *p*-value < 0.05. We found 342 genes were upregulated in the 5 mg/kg/d group compared with the control group, among which the most significant gene was “RT1 class I, locus CE2 (RT1-CE2)", and the difference multiple was about 8.41 (*p*<0.001). (Figure 6A). The clustering effect of differentially expressed genes between the two groups was significant (Figure 6B). KEGG enrichment analysis identified the steroid biosynthesis pathway (*p* = 1.940, FDR<0.01) and ascorbate and aldarate metabolism (*p* < 0.01, FDR = 0.020) pathways (Figure 6C). There were 19 genes enriched in the steroid biosynthetic pathway, including 7 upregulated genes, such as lanosterol synthase (LSS) (*p* < 0.001), NAD(P)-dependent steroid dehydrogenase-like (NSDHL) (*p* < 0.05), sterol-C5 desaturase (SC5D) (*p* < 0.01), methylsterol monooxygenase 1 (MSMO1) (*p* < 0.05), cytochrome P450 family 51 (CYP51) (*p* < 0.01), transmembrane 7 superfamily member 2 (TM7SF2) (*p* < 0.01) and squalene epoxidase (SQLE) (*p* < 0.001) (Figure 7). The rate-limiting enzyme SQLE and monooxygenase CYP51 in steroid biosynthesis were selected for further verification. IHC results showed that compared with the control group, the expression of CYP51 (*p* < 0.001) and SQLE (*p* < 0.01) in ovaries of rats in 5 mg/kg/d group increased significantly, but there was no significant change in the expression of CYP51 and SQLE in uterus of rats (*p* > 0.05). (Figures 8A–C). The qPCR results showed that the mRNA expression levels of CYP51 (20.86 ± 0.55) and SQLE (21.85 ± 0.32) in the 5 mg/kg/d group were significantly higher than those in the control group (19.94 ± 0.70, 20.63 ± 0.51) (*p* < 0.05), but there was no significant difference in the mRNA expression levels of RT1-CE2 (*p* > 0.05) (Figure 9).

## Discussion

### Correlation between phthalates and endometriosis

In the current analysis of our clinical data, we found that every one-unit increase in urine the concentration of DMP, MMP, DEP, MEP, DBP, MBP, or DEHP increased the risk of endometriosis by 1.3–8.5 times. Wei C. et al. found that mono (2-ethyl-5-hydroxyhexyl) phthalate (MEHHP) exposure may also be a risk factor for endometriosis (OR = 1.2, 95% CI = 1.0–1.5) (Cai et al., 2019). However, Kristen U. et al. proposed that the increase in the concentration of MBzP or MEP in urine can increase the risk of endometriosis but that the concentrations of DEHP, MEHP, and MEHHP were even negatively correlated with the risk of endometriosis (Upson et al., 2013). The different types and levels of phthalate metabolite exposure among people in different regions may be related to this outcome. In this study, 33.3% of endometriosis patients had a failed pregnancy after surgery, and the rate of IVF-ET among patients with successful pregnancy was also higher. In women with endometriosis, reduced ovarian reserve is manifested by a significant decrease in oocyte count and AMH levels (Collodel et al., 2023). The concentration of DEHP in urine is negatively correlated with the number of oocytes, clinical pregnancy and live birth (Hauser et al., 2016). Encircling granulosa cells can protect oocytes from DEHP-induced apoptosis, and the existence of granulosa cells can play a positive role in the survival of oocytes *in vitro*, which may be helpful in assisted reproductive technology (Tripathi et al., 2019). Surgical combined assisted reproductive techniques have been shown to offer a higher chance of pregnancy for infertile women with endometriosis. However, pelvic surgery for endometriosis, especially for endometrioma, may cause iatrogenic injury due to loss of ovarian reserve function, adhesion formation and ischemic injury (Coccia et al., 2022).

We recommend that women adopt a healthy lifestyle and minimize their exposure to cosmetics and plastics, which can increase their exposure levels to phthalates and cause damage to reproductive function. In our study, we also analysed the correlation between 10 phthalate metabolites and endometriosis stage, infertility history, reproductive prognosis, postoperative recurrence and dysmenorrhea, but there were no significant associations. However, other studies have suggested that the serum levels of MBzP, MEHHP, DBP, BBzP, and DEHP in women with endometriosis and infertility are higher and that the serum phthalate content is strongly correlated with endometriosis severity (Reddy et al., 2006; Pednekar et al., 2018). Sadia N. et al. found that the average serum DEHP concentration of women with endometriosis was 65.29 ± 21.69 ng/mL, which was significantly higher than that of the controls, and the serum DEHP concentration of women with advanced endometriosis (III and IV) had an increasing trend (Nazir et al., 2018). This may be related to our insufficient sample size. To further clarify this result, the sample size will need to be increased, or a multicentre cohort study will need to be conducted.

In clinic, ovarian dysfunction and infertility in patients with endometriosis have attracted much attention. The decrease of ovarian reserve caused by endometrioma is a major problem for infertile women related to endometriosis. The existence of endometrioma focus is one of the reasons for the decrease of ovarian reserve, and the resection of endometriosis cyst may destroy the normal tissue structure of ovarian cortex, which will have a negative impact on postoperative ovarian reserve and affect the treatment results of endometrioma-related infertile women (Kitajima et al., 2018). Peritoneal endometriosis may affect the follicular microenvironment by inducing macrophage recruitment, cytokine release and reactive oxygen species production, and inflammatory changes in peritoneal fluid may affect sperm function and increase the incidence of female infertility (Broi et al., 2019). However, other studies have shown that there is no significant difference in AMH level between women with peritoneal endometriosis and normal women (Goodman et al., 2016). The effect of peritoneal endometriosis on ovarian reserve function in women is still unclear. Assessment of ovarian reserve may play an important role in the management of reproductive health in women with endometriosis. Exposure to phthalates in women with endometriosis may further aggravate the reduction of ovarian reserve. The correlation between endometriosis and phthalates has been confirmed in our study, but it is not clear whether the decline of fertility in endometriosis patients is related to the decline of ovarian function after phthalates exposure. Exploring the effect of phthalates on ovarian function is of great significance to human fertility. DEHP is the main form of phthalate in nature (Sheikh, 2016), which has a typical prediction for the diagnosis of endometriosis. Therefore, we chose DEHP for further animal experiments, to explore the effects of different doses of DEHP exposure on ovarian function in rats, and to preliminarily explore the possible mechanisms of genes and signal transduction pathways related to ovarian function damage caused by phthalates.

### Effect of DEHP on ovarian function and its mechanism

In our animal experiments, DEHP exposure did not have a significant effect on the weight gain of rats; however, the oestrous cycle was disrupted, and the serum AMH levels and uterine and ovarian organ coefficients were all reduced in a dose-dependent manner. It is worth noting that in the 5 mg/kg/d treatment group, we did not observe significant changes in serum AMH level and uterine and ovarian organ coefficients, which may be because the exposure dose of this treatment group corresponds to the human safety limit value of DEHP. Seul et al. showed that cysts formed from ectopic endometrial tissue have potential toxic effects on the development of preantral follicles in mice and damage the function of adjacent ovarian tissues (Kim et al., 2018). Under exposure to phthalates, follicular consumption will further increase, and preantral follicular atresia will be aggravated (Vabre et al., 2017). Polystyrene microplastics can cause apoptosis and fibrosis of granular cells in rat ovaries through oxidative stress, reduce the number of follicles, and significantly reduce serum AMH levels (An et al., 2021). Compared to healthy women, patients with endometriosis had lower AMH and sinus follicle counts, especially in patients with endometriosis (III and IV), with a more significant decrease in AMH levels and a reduced number of oocytes and transferable embryos obtained (Safdarian et al., 2018; Feferkorn et al., 2023). Phthalates, a high-production industrial chemical used in the manufacture of plastics, expose women to higher levels than men, disrupting female follicle growth patterns and causing premature follicle apoptosis, which leads to more rapid failure of ovarian reserves and the onset of infertility (Panagiotou et al., 2021). Therefore, it is necessary to detect AMH level in time to evaluate the severity of ovarian endometriosis and the possibility of successful pregnancy after operation. After DEHP exposure, the level of gonadotropin-releasing hormone in the rat hypothalamus increased, the level of gonadotropin-releasing hormone receptor mRNA and protein in the pituitary increased, and the expression level of luteinizing hormone receptor was downregulated (Liu et al., 2014; Liu et al., 2016; Qin et al., 2019). DEHP may lead to the destruction of estrogen biosynthesis pathway and the imbalance of hypothalamus-pituitary-ovary axis in female rats, which has a negative impact on the development and function of reproductive system (Liu et al., 2014). Deborah et al. found that starting from the 50 mg/kg/d DEHP exposure level, the oestrogen level of rats during oestrus decreased, the follicle-stimulating hormone level increased significantly, and the thickness of the follicular membrane cell layer decreased. When rats are exposed to DEHP at a dose of 20 mg/kg/d, their F3 pregnancy rate and birth weight are reduced, while the surge of steroids during oestrus is reduced (Meltzer et al., 2015).

According to the actual dietary pattern, the total dietary intake of DEHP for women of childbearing age is 5.7 μg/kg/day (Serrano et al., 2014), which is far below the safe limit value of human body, but it is not excluded that DEHP enters the human body in other ways, such as respiratory inhalation and skin contact. The work of Hannon et al. has also enhanced our understanding of DEHP effect. They have clarified that short-term and low-level exposure will lead to abnormal ovarian function, and the obvious toxicity of very high dose may not be a necessary part of the harmful effects on ovaries (Hannon et al., 2014). Therefore, we choose the lowest dose group (5 mg/kg/d treatment group) of rat ovarian tissue for bioinformatics analysis. Although in the 5 mg/kg/d treatment group, we did not observe the significant changes of serum AMH level and uterine and ovarian organ coefficients, but the estrous cycle and biological information analysis showed valuable results. In the bioinformatics analysis, regarding the possible effects of DEHP on rat ovarian function, we initially screened the steroid biosynthesis pathway and ascorbate aldarate metabolism, the latter of which is used mostly in plant-related research. It is worth noting that the steroid biosynthetic pathway starts with cholesterol synthesis and can produce steroid hormones in the adrenal gland, testis, ovary, and placenta and participate in the production of ovarian oestrogen under the action of aromatase. DEHP can interfere with ovarian function through the 17β-hydroxysteroid dehydrogenase (17β-HSD) signalling pathway, prolong the oestrus cycle, increase follicular atresia, reduce the expression of steroid-producing enzymes, and promote the apoptosis of ovarian granulosa cells (Li et al., 2020). Steroid hormone signalling-related genes such as LSS, SQLE, MSMO1, and NSDHL are involved in the development of ovarian follicles (Bunel et al., 2014; Li et al., 2020). SQLE may be a regulatory gene related to steroid biosynthesis and inflammation, and it can reflect the changes in granulosa cells in patients with ovarian hypofunction (He et al., 2020). Although “RT1-CE2” is a mouse-derived gene, it contains a series of characteristic non-classical major histocompatibilty complex (MHC) molecules, which may be related to immune inflammatory response (Dai et al., 2018), which may provide a direction for our future research on the population. CYP51 is a key enzyme involved in sterol and steroid biosynthesis in follicular development and oocyte maturation, thyroid hormone and gonadotropin levels have a regulatory effect on the process of follicle formation and oocyte maturation involved in CYP51 (Weng et al., 2019)^.^ In addition, other studies have found that the abnormal expression of Cytochrome P450 Family 19 Subfamily A Member 1 (CYP19A1), Epidermal Growth Factor Receptor (EGFR), Estrogen Receptor 2 (ESR2), Fos Proto-Oncogene, AP- 1 Transcription Factor Subunit (FOS), and Insulin-like growth factor 1 (IGF1) related to the steroid pathway may also be related to the ovarian dysfunction of endometriosis (Roy et al., 2015) It is noteworthy that the genetic changes in ovaries are at least partly due to steroid biosynthesis. DEHP may affect the production of estrogen in ovary by changing the expression of genes related to steroid biosynthesis pathway, which will eventually promote the development of endometriosis.

There were also some limitations in this study. This study reflects mainly the short-term phthalates exposure level of the local female population, and its test results cannot represent the long-term phthalates exposure of the population or exposure in other regions or ethnic groups. In addition, the animal experiments did not include a long-term DEHP exposure group. Whether DEHP is time-dependent on rat ovarian function needs to be explored.

## Conclusion

Endometriosis is a disease of the gene‒environment‒endocrine interaction. The metabolites of phthalates in urine, such as DMP, MMP, DEP, MEP, DBP, MBP, BBzP, DEHP and MEHP, are related to the prevalence of endometriosis, while DEHP has the highest risk for endometriosis. Frequently using plastic products and cosmetics may increase the exposure level of phthalates. Finally, the steroid biosynthesis pathway is related to ovarian dysfunction in rats exposed to DEHP.

## Data Availability

The datasets presented in this study can be found in online repositories. The names of the repository/repositories and accession number(s) can be found below: BioProject: PRJNA931580.
